# The Degree of Extramural Spread of T3 Colon Cancer as a Prognostic Factor: Another Appeal to the American Joint Committee on Cancer

**DOI:** 10.1002/cam4.70720

**Published:** 2025-04-29

**Authors:** Beatriz Arencibia‐Pérez, Francisco Giner, Eduardo García‐Granero, Susana Roselló‐Keränen, Blas Flor‐Lorente, Andrés Cervantes, Jorge Sancho‐Muriel, Matteo Frasson

**Affiliations:** ^1^ Colorectal Surgery Unit Hospital Universitari i Politècnic La Fe, University of Valencia València Spain; ^2^ Department of Pathology Hospital Universitari i Politècnic La Fe, University of Valencia València Spain; ^3^ Department of Medical Oncology Hospital Clínico Universitario, University of Valencia València Spain

**Keywords:** cancer, colon, infiltration, pT3, recurrence, survival

## Abstract

**Background:**

The pT3 category of colon cancer staging is heterogeneous and has significant prognostic value. However, this is not reflected in the current TNM staging system. The objective of this work is to determine whether the extent of infiltration beyond the *muscularis propria* of pT3 colon carcinoma is an independent risk factor for worse oncologic outcomes after curative surgery.

**Methods:**

Retrospective analysis of 536 patients from a tertiary University Hospital with pT3M0 colon cancer (1995–2015) was collected and re‐evaluated to assess tumor infiltration extent beyond the *muscularis propria* layer. The main outcome measures studied were local recurrence, systemic recurrence, disease‐free survival, and cancer‐specific survival.

**Results:**

An infiltration extent of 5 mm was the best cutoff for predicting oncological results in this group of patients. Multivariable analysis showed that tumor infiltration depth into the pericolic fat was an independent risk factor for a higher local recurrence rate (*p* = 0.02, HR 1.11 per mm, 95% CI 1.04–1.23), a higher risk of systemic recurrence (*p* = 0.02, HR 1.08 per mm, 95% CI 1.01–1.16), worse disease‐free survival (*p* = 0.008, HR 1.08 per mm, 95% CI 1.02–1.14), and cancer‐specific survival (*p* = 0.009, HR 1.09 per mm, 95% CI 1.02–1.16). In a sub‐analysis, these results were confirmed in patients with positive lymph nodes but not in the group of patients with negative lymph nodes.

**Conclusions:**

The extramural spread of pT3 colon cancer is a significant prognostic factor for worse oncological outcomes after curative surgery. Therefore, this parameter should be considered in selecting adjuvant therapy and possibly included in the TNM staging system.

## 1 Introduction

Colorectal tumors categorized as pT3 refer to invasive tumors that penetrate beyond the *muscularis propria* into the subserosal adipose tissue or non‐peritonealized pericolic or perirectal tissues [1]. This category encompasses 60%–70% of colorectal cancers [2, 3]. Despite being viewed as a homogeneous group, pT3 tumors exhibit significant heterogeneity in oncological outcomes due to varying degrees of invasion beyond the *muscularis propria* layer.

In rectal cancer, several studies have demonstrated the clinical relevance of subclassifying pT3 tumors based on the extent of tumoral spread beyond the *muscularis propria* [4, 5, 6, 7, 8, 9]. These studies often classify pT3 tumors into two subgroups: pT3a or pT3b, based on extramural spread ≤ 5 or > 5 mm, respectively [8, 9]. Tumors classified as pT3b are associated with a significantly higher incidence of nodal involvement, venous invasion, distant metastasis, and worse oncologic outcomes, including recurrence and survival rates. This subclassification has been proposed to guide adjuvant therapy planning [6, 10, 11]. Furthermore, it has potential implications for preoperative prognostic assessment and selection of neoadjuvant therapy in cT3 rectal cancer staging, particularly when utilized alongside accurate MRI and circumferential margin status evaluation [8, 12, 13].

Conversely, limited specific information exists regarding potential ramifications of pT3 classification in colon cancer. Merkel et al. [14] initially proposed infiltration depth > 15 mm as a risk factor for patients with Stage II colon carcinoma, recommending adjuvant chemotherapy for such cases. However, the same group [15] has recently suggested subdividing pT3 into T3a (≤ 1 mm), T3b (> 1–15 mm), and T3c (> 15 mm). Other recent studies in colon cancer have proposed different cutoff values for worse prognosis, such as 5 mm [16] or 3 mm [17], although the latter study includes both rectal and colon cancer. Notably, none of these studies have properly analyzed the effect of this subclassification on local recurrence rates.

Initially proposed in the TNM (3rd edition) Supplement [18], a subdivision of pT3 for all colorectal cancers into four grades—pT3a (< 1 mm), pT3b (1–5 mm), pT3c (5–15 mm), and pT3d (> 15 mm)—was based on infiltration depth into the extramural fat. However, the most recent TNM (5th edition) Supplement [19] maintains the same subdivision of pT3 for colonic cancers based exclusively on data from the Erlangen Registry Cancer (1986–2003), while proposing a dichotomous subdivision for rectal cancers—pT3a and pT3b—depending on tumor invasion beyond the *muscularis propria*.

Despite the accumulating evidence and proposals, the subdivision of pT3 has not been incorporated yet into the TNM colorectal cancer staging system [20]. Therefore, further studies supporting the clinical relevance of subdividing pT3 colon tumors based on infiltration extent, especially regarding local recurrence rates, are warranted to advocate for its inclusion.

This study aims to ascertain whether the depth of pT3 infiltration beyond the *muscularis propria*, measured in millimeters, represents an independent risk factor for worse oncologic outcomes following curative colon cancer resection.

## 2 Material and Methods

### 2.1 Patients

This is a retrospective study including data from a prospective institutional database from a Colorectal Unit in a tertiary university hospital with a specific multidisciplinary team for colorectal cancer treatment. The study includes a consecutive series of patients with pT3M0 colon cancer (without hereditary syndromes or inflammatory bowel disease) who underwent curative surgery between 1995 and 2015. Patients with unavailable pathological samples for microscopic re‐evaluation were excluded. Samples (usually 5–6 from each patient) were re‐evaluated by a pathologist (F.G.) specialized in colorectal cancer, unaware of the patients' clinical data. The study was approved by the Biomedical Investigation Ethics Committee.

The depth of infiltration into the extramural pericolic tissues beyond the *muscularis propria* was measured microscopically in millimeters using an optic microscope (Leica, DMD 108) and the Vernier scale. When the outer border of the muscular layer was not entirely identifiable due to invasion, an estimate of the outer border was obtained between both break points in the *muscularis propria*. Only the deepest extent of continuous invasion was noted; foci of tumor satellites and metastatic lymph nodes in close proximity to the primary were not considered.

Elective surgical procedures were always performed or supervised by a colorectal surgeon. Urgent surgeries were performed by the on‐call staff, consisting of general or colorectal surgeons. In block colon cancer resection was determined by the tumor site and lymphovascular drainage, according to the standardized technique described previously by other authors [21, 22]. The concept of standardized resections with CME and central tie of the artery supply was formally introduced in our department in 2009 [3].

### 2.2 Outcome Assessment

All patients were discussed by a multidisciplinary team, including the decision for adjuvant chemotherapy adapted to the individual clinical situation and staging. The follow‐up protocol has been described previously [23], and two oncologists (AC, SR) within the MDT acted as independent observers to confirm the presence of disease recurrence. The term recurrence is defined as the local or systemic appearance of tumor cells derived from the primary malignancy, following curative surgery. As local recurrence, we have used the criteria used in scientific articles by Bertelsen CA et al. [24]; Bowne WB et al. [25]; Reed et al. [26], Bokey L et al. [27]; Harris GJC et al. [28]; Manfredi S et al. [2]; Baguena G et al. [23], where any oncological finding in the suture line or anastomosis, in the area of the previous tumor, in the area of resection of the lymph node drainage, in neighboring organs or structures, and/or in the peritoneum with carcinomatosis was included as local recurrence.

The main outcome measures considered were local recurrence (LR), defined as the period between surgery and the appearance of local recurrence; systemic recurrence (SR), defined as the period between surgery and the appearance of distant metastases; disease‐free survival (DFS), defined as the period between surgery and the appearance of either local or systemic recurrence; and cancer‐specific survival (CSS), defined as the period between surgery and death due to recurrent locoregional carcinoma and/or distant metastases.

### 2.3 Statistical Analysis

Categorical variables are expressed as absolute frequencies and percentages, and quantitative variables are expressed as median and interquartile range. To evaluate the best cut‐off for the infiltration depth as a prognostic factor for oncological outcomes, we analyzed the receiver operating characteristic (ROC) curves and calculated the area under the curve (AUC). The best cut‐off for infiltration depth (mm) into the pericolic tissues beyond the *muscularis propria* for predicting LR, SR, DFS, and CSS was determined using the Youden index, and the patients were classified in three groups according to this cut‐off and double its value. Oncological results (LR, SR, DFS, and CSS) were represented by Kaplan–Meier curves. The univariate influence of the depth of pericolic infiltration and other pathological variables on LR, SR, DFS, and CSS was analyzed with the Kaplan–Meier method and the log‐rank (Mantel‐Cox) test. In addition to tumor infiltration depth, multiple variables were analyzed: age (continuous), sex, tumor location, degree of differentiation, histological type, lymphatic/vascular/perineural invasion, tumor perforation, type of surgery, and adjuvant treatment.

A Cox multivariate regression model was then constructed, including variables with *p* < 0.10 at univariate analysis. The proportional hazards assumption of the Cox model was assessed. Moreover, the same model was repeated, introducing the variable depth of invasion as a continuous variable, allowing an evaluation of the risk per mm of invasion.

Finally, a sub‐analysis (univariate Cox regression) including only pN0 or pN+ patients was performed to explore the association of the depth of invasion with oncological outcomes in these subgroups of patients.

Statistical significance was defined as *p* < 0.05. The statistical data were processed using the Statistical Package for the Social Sciences (SPSS, version 21.0.0).

Data were reported according to the STROBE checklist [29].

## 3 Results

### 3.1 Study Population

**FIGURE 1:**
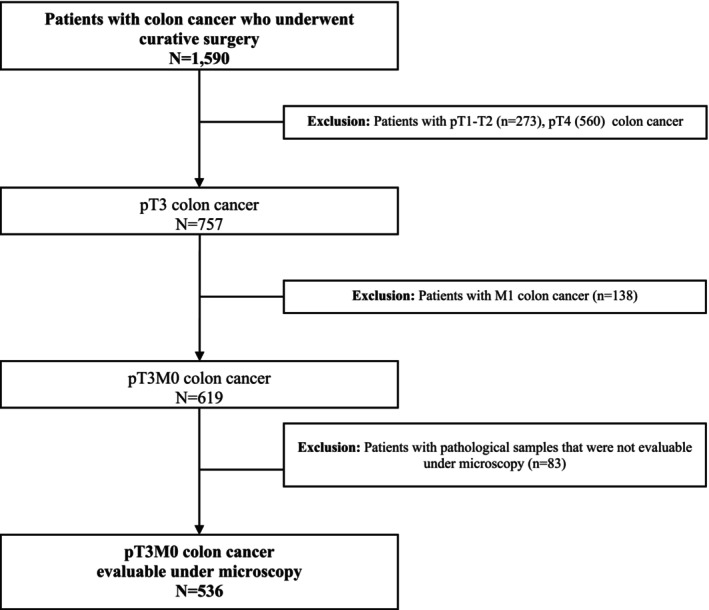
Flow chart of patients in the study.

From 1995 to 2015, 1590 patients were included in the prospective database, and 536 of them fulfilled the inclusion and exclusion criteria and were analyzed in the present study (Figure 1). Most of the patients had pN0 (61.3%) and moderately differentiated tumors (62.8%). The median follow‐up for the whole group was 68.0 months (25th–75th percentile: 43–101 months). Demographic and clinical characteristics of patients are shown in Table 1. The median infiltration extent beyond the *muscularis propria* was 3.5 mm (25th–75th percentile: 2.0–6.0 mm). Tables 2, 3, 4, 5 show the results of the multivariate analysis for LR, SR, DFS, CSS.

**TABLE 1:** Demographic and clinical characteristics of patients. Data are reported as the number of patients (%) or median (25th–75th percentile).

	Patients (*N* = 536)
Age (years)	73 (64–79)
Sex, *n* (%)	
Male	287 (53.5)
Female	249 (46.5)
N stage—lymph nodes involvement, *n* (%)	
pN0	333 (62.1)
pN1	140 (26.1)
pN2	63 (11.8)
Number of lymph nodes examined	13 (8–22)
Location *n* (%)	
Right colon	181 (33.8)
Transverse colon	52 (9.7)
Left colon	303 (56.5)
Differentiation degree, *n* (%)	
Well differentiated	109 (20.3)
Moderately differentiated	333 (62.1)
Poorly differentiated	90 (16.8)
Undifferentiated	4 (0.7)
Histological type, *n* (%)	
Conventional	441 (82.2)
Mucinous features (< 50% of mucin)	50 (9.3)
Mucinous subtype (> 50% of mucin)	38 (7.1)
Signet ring cell	7 (1.3)
Tumor infiltration, *n* (%)	
Lymphatic	147 (27.4)
Vascular	169 (31.5)
Perineural	129 (24.1)
Tumor perforation, *n* (%)	
Absent	509 (95.0)
Spontaneous	25 (4.7)
Iatrogenic	2 (0.3)
Type of surgery, *n* (%)	
Elective	409 (76.3)
Urgent	134 (23.7)
Adjuvant treatment, *n* (%)	200 (37.3)

Abbreviation: *N*, number of cases.

**TABLE 2:** Multivariate analysis of risk factors for local recurrence (LR).

	Multivariate analysis		
	Hazard ratio (95% CI)	*p*	5‐year local recurrence rate (%)
Infiltration depth (ID)		**0.02**	
< 5 mm	1		3.2
5–10 mm	2.9 (1.3–6.8)		12.2
> 10 mm	3.6 (1.02–12.9)		14.9
N stage—lymph nodes involvement		0.4	
pN0	1		3.7
pN1	1.6 (0.7–3.9)		10.5
pN2	2.0 (0.7–5.8)		11.3
Differentiation degree		0.4	
Well differentiated	1		4.1
Moderately differentiated	0.9 (0.3–2.9)		4.3
Poorly differentiated/undifferentiated	1.9 (0.5–6.5)		8.0
Histological type		0.7	
Conventional	1		5.5
Mucinous features (< 50% of mucin)	1.4 (0.5–4.3)		9.2
Mucinous subtype (> 50% of mucin)	0.7 (0.1–5.1)		3.2
Signet ring cell	2.1 (0.4–10.4)		28.6
Tumor infiltration			
Lymphatic	1.4 (0.6–3.1)	0.4	9.7
Tumor perforation			19.0

*Note:* Bold values indicates statistical significance.

Although all the variables indicated were explored, only those that showed statistical significance in the univariate analysis and that were also selected for the multivariate analysis are included in the tables presented.

The actuarial 5‐year rates for LR, SR, DFS, and CSS were 6.3%, 15.5%, 78.3%, and 83.8%, respectively.

### 3.2 Extent of Extramural Spread and Oncologic Outcomes

**FIGURE 2:**
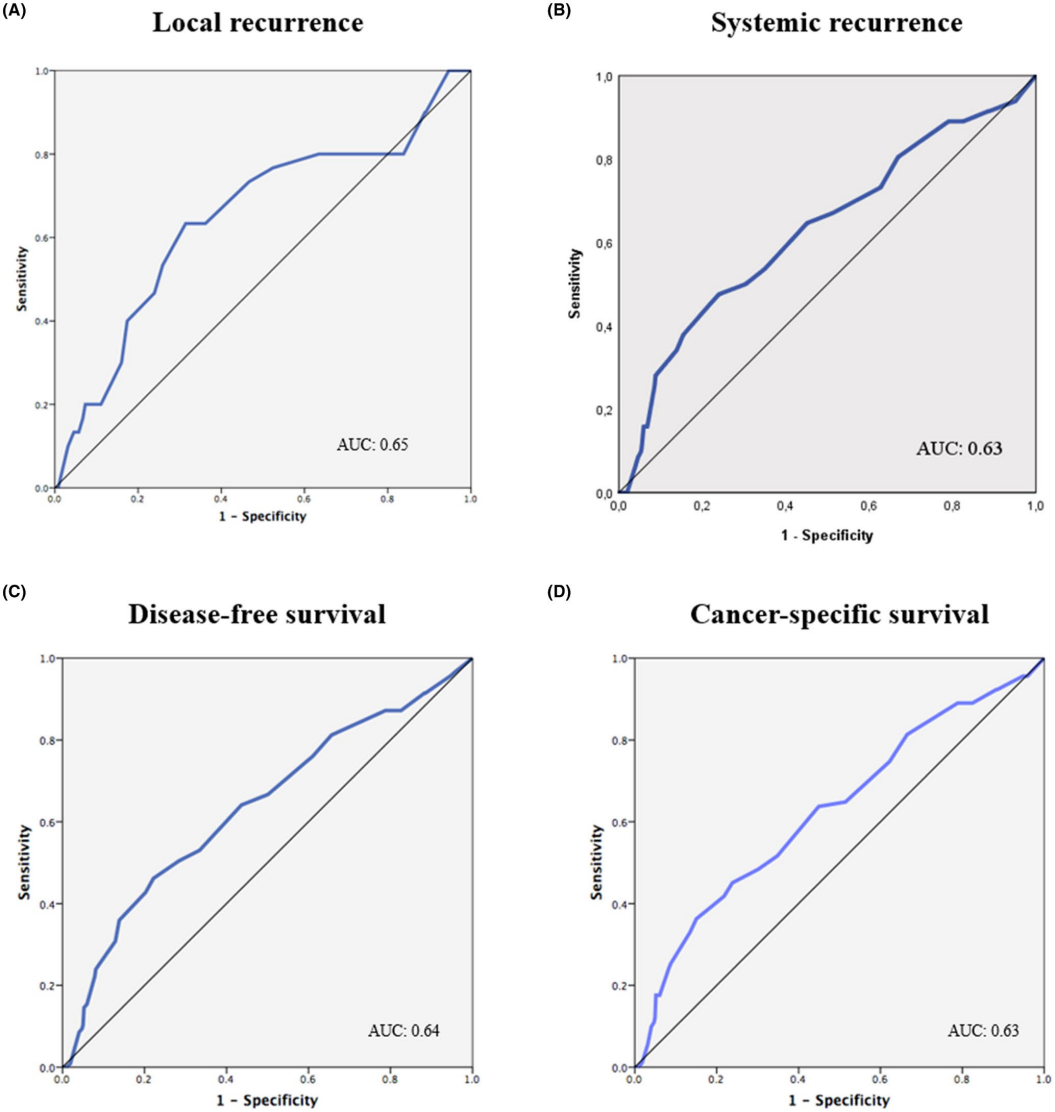
Receiver Operating Characteristic (ROC) curves on infiltration depth (mm) for predicting local recurrence (A), systemic recurrence (B), disease‐free survival (C) and cancer‐specific survival (D).

The AUC for infiltration extent was 0.65 for predicting local recurrence, 0.63 for systemic recurrence, 0.64 for overall recurrence, and 0.63 for cancer‐related death (Figure 2). An infiltration extent of 5 mm was the best cut‐off for predicting local recurrence (63.3% sensitivity and 68.6% specificity), systemic recurrence (50.0% sensitivity and 69.6% specificity), DFS (50.4% sensitivity and 71.6% specificity), and CSS (45.1% sensitivity and 76.2% specificity). Therefore, patients were classified according to the 5 mm and 10 mm cut‐offs: ≤ 5 mm (66.8% of patients), 6–10 mm (27.4%), and > 10 mm (5.8%).

**FIGURE 3:**
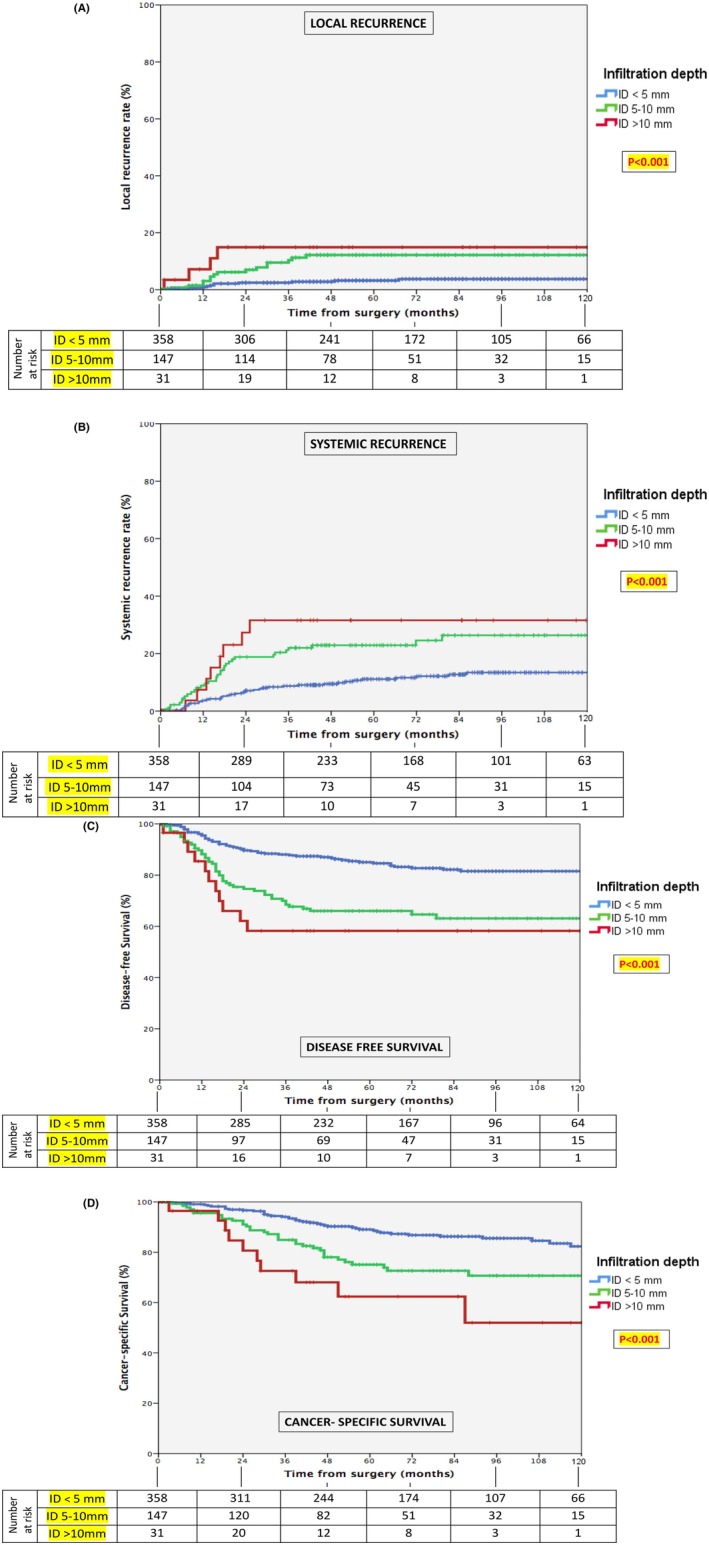
Survival curves for local recurrence (A), systemic recurrence (B), disease‐free survival (C) and cancer‐specific survival (D) according to the depth of tumor infiltration into the mesenteric fat. ID, infiltration depth.

According to this sub‐classification, the extent of infiltration was significantly associated with local recurrence (*p* < 0.001), systemic recurrence (*p* < 0.001), DFS (*p* < 0.001), and CSS (*p* < 0.001) at univariate analysis (Figure 3).

At multivariate Cox regression analysis, compared with patients having an infiltration extent ≤ 5 mm, those with 6–10 mm showed 2.9 (95% CI: 1.3–6.8), 1.7 (95% CI: 1.1–2.8), 1.9 (95% CI: 1.3–2.9), and 1.6 (95% CI: 1.01–2.5) greater likelihoods for higher LR, higher SR, worse DFS, and worse CSS, respectively. Similarly, patients with an infiltration extent > 10 mm showed 3.6 (95% CI: 1.02–12.9), 1.9 (95% CI: 0.9–4.4), 2.0 (95% CI: 1.01–4.1), and 2.4 (95% CI: 1.2–5.0) higher probabilities for higher LR, higher SR, worse DFS, and worse CSS, respectively (Tables 2, 3, 4, 5).

No other variables were associated with LR at multivariable analysis (Table 2), while N stage (*p* = 0.001), vascular infiltration (*p* = 0.04), and urgent surgery (*p* = 0.046) were independent risk factors for SR (Table 3), and N stage (*p* < 0.001) and urgent surgery (*p* = 0.006) were independent risk factors for worse DFS (Table 4). Along with the degree of extramural spread, N stage (*p* < 0.001), vascular invasion (*p* = 0.04), and urgent surgery (*p* = 0.03) were independent risk factors for worse CSS at multivariable analysis (Table 5).

**TABLE 3:** Multivariate analysis of risk factors for systemic recurrence (SR).

	Multivariate analysis		
	Hazard ratio (95% CI)	*p*	5‐year systemic recurrence rate (%)
Infiltration depth (ID)		**0.03**	
< 5 mm	1		11.1
5–10 mm	1.7 (1.1–2.8)		22.9
> 10 mm	1.9 (0.9–4.4)		31.6
N stage—lymph nodes involvement		**0.001**	
pN0	1		7.8
pN1	2.4 (1.4–4.2)		16.8
pN2	3.3 (1.7–6.4)		35.3
Differentiation degree		0.9	
Well differentiated	1		11.4
Moderately differentiated	1.0 (0.5–1.9)		15.2
Poorly differentiated/Undifferentiated	1.1 (0.5–2.3)		21.8
Tumor infiltration			
Lymphatic	1.0 (0.6–1.7)	0.9	22.1
Vascular	1.8 (1.04–3.3)	**0.04**	27.2
Perineural	0.8 (0.5–1.5)	0.5	23.8
Type of surgery		**0.046**	
Scheduled	1		12.3
Urgent	1.6 (1.01–2.6)		23.4
Adjuvant treatment	1.3 (0.8–2.1)	0.3	23.3

*Note:* Bold values indicates statistical significance.

**TABLE 4:** Multivariate analysis of risk factors for disease‐free survival (DFS).

	Multivariate analysis		
	Hazard ratio (95% CI)	*p*	5‐year disease‐free survival rate (%)
Infiltration depth (ID)		**0.004**	
< 5 mm	1		85
5–10 mm	1.9 (1.3–2.9)		66
> 10 mm	2.0 (1.01–4.1)		58
N stage—lymph nodes involvement		**< 0.001**	
pN0	1		88
pN1	2.3 (1.4–3.6)		64
pN2	3.1 (1.8–5.6)		56
Differentiation degree		0.6	
Well differentiated	1		85
Moderately differentiated	1.1 (0.1–8.5)		80
Poorly differentiated/Undifferentiated	1.52 (0.2–11.9)		61
Histological type		0.4	
Conventional	1		79
Mucinous features (< 50% of mucin)	1.3 (0.7–2.3)		69
Mucinous subtype (> 50% of mucin)	0.5 (0.1–1.5)		91
Signet ring cell	1.5 (0.4–5.1)		57
Tumor infiltration			
Lymphatic	1.1 (0.7–1.8)	0.5	69
Vascular	1.4 (0.8–2.3)	0.2	66
Perineural	0.8 (0.5–1.4)	0.4	69
Type of surgery		**0.006**	
Scheduled	1		82
Urgent	1.7 (1.2–2.6)		67
Adjuvant treatment	1.1 (0.7–1.47)	0.7	69

*Note:* Bold values indicates statistical significance.

**TABLE 5:** Multivariate analysis of risk factors for cancer‐specific survival (CSS).

	Multivariate analysis		
	Hazard ratio (95% CI)	*p*	5‐year cancer‐specific survival rate (%)
Infiltration depth (ID)		**0.02**	
< 5 mm	1		89
5–10 mm	1.6 (1.01–2.5)		75
> 10 mm	2.4 (1.2–5.0)		62
N stage—lymph nodes involvement		**< 0.001**.	
pN0	1		92
pN1	2.8 (1.7–4.8)		75
pN2	4.9 (2.6–9.4)		58
Differentiation degree		0.5	
Well differentiated	1		86
Moderately differentiated	0.97 (0.5–1.8)		86
Poorly differentiated/Undifferentiated	1.4 (0.7–2.9)		72
Tumor infiltration			
Lymphatic	0.8 (0.5–1.3)	0.4	77
Vascular	1.8 (1.03–3.2)	**0.04**	72
Perineural	0.8 (0.5–1.5)	0.6	74
Type of surgery		**0.03**	
Scheduled	1		86
Urgent	1.6 (1.1–2.6)		76
Adjuvant treatment	0.8 (0.5–1.2)	0.3	80

*Note:* Bold values indicates statistical significance.

When considering the extramural infiltration extent as a continuous variable in the same multivariate Cox regression model, it was confirmed to be an independent risk factor for a higher risk of local recurrence (*p* = 0.04, HR 1.11 per mm), a higher risk of SR (*p* = 0.02, HR 1.08 per mm), worse DFS (*p* = 0.008, HR 1.08 per mm) and worse CSS (*p* = 0.009, HR 1.09 per mm).

The percentage of patients that were treated with adjuvant therapy was 34.3% (depth on infiltration < 5 mm), 44.2% (5–10 mm) and 53.6% (> 10 mm).

When performing a subset analysis based on the presence of lymph node involvement, in the pN+ group, the depth of invasion was significantly associated with local recurrence (*p* = 0.015), systemic recurrence (*p* < 0.001), disease‐free survival (*p* < 0.001), and cancer‐specific survival (*p* < 0.001). However, when considering only pN0 patients, this association was not statistically significant (minimum *p* = 0.08) (Table 6).

**TABLE 6:** 5‐year actuarial rate for local recurrence (LR), systemic recurrence (SR), disease free survival (DFS) and cancer‐specific survival (CSS) according to the depth of extra mural invasion, stratified for pN status.

	pN‐ (*n* = 333)				pN+ (*n* = 203)				All patients (*n* = 536)			
	< 5 mm (*n* = 268)	5–10 mm (*n* = 55)	> 10 mm (*n* = 10)	*p*	< 5 mm (*n* = 120)	5–10 mm (*n* = 65)	> 10 mm (*n* = 18)	*p*	< 5 mm (*n* = 388)	5–10 mm (*n* = 120)	> 10 mm (*n* = 28)	*p*
LR	3.0%	8.1%	0.0%	0.25	6.0%	16.2%	24.7%	0.015	3.9%	12.1%	16.2%	0.001
SR	7.3%	11.6%	0.0%	0.31	19.6%	42.2%	50.0%	< 0.001	10.8%	27.2%	31.2%	< 0.001
DFS	89.8%	80.5%	100.0%	0.08	74.9%	44.3%	31.3%	< 0.001	85.5%	61.7%	54.6%	< 0.001
CSS	92.5%	85.8%	100.0%	0.57	80.4%	59.1%	46.2%	< 0.001	89.0%	71.9%	58.8%	< 0.001

## 4 Discussion

The present analysis confirms the prognostic heterogeneity of pT3 colonic tumors according to the extramural soft tissue invasion beyond the *muscularis propria*, which is an independent risk factor for LR, SR, and is associated with worse DFS and CSS. These data indicate that the deeper a tumor invades into the extramural tissues, the worse the prognosis is, and the best cut‐off value is more than 5 mm of tumor infiltration that predicts significantly higher local and systemic recurrences, and worse survival.

This is in agreement with most rectal cancer studies concluding that an infiltration depth > 5 mm outside the *muscularis propria* has an adverse prognostic value and might be useful for predicting patient outcomes and selecting adequate treatments [8, 9].

There is scarce and controversial information in colon cancer specifically analyzing the prognosis of tumoral extent beyond the muscular layer in pT3 tumors [14, 15, 16, 17]. Similarly to some of the most recent studies, we observed significantly worse LR, SR, DFS, and CSS outcomes depending on the extent of infiltration. Moreover, in the present study, this association was confirmed in the group of patients with positive lymph nodes, but not in those with negative lymph nodes. This finding is in agreement with the last series published by Merkel et al. [15], and the results of Macchi et al. [16] and Foersch et al. [17].

There exists a lack of uniform criteria regarding how to subclassify pT3 tumors. In the last study of Merkel et al. [15], they arbitrarily subdivide pT3 into three subgroups: pT3a (≤ 1 mm), pT3b (1–15 mm), and pT3c (> 15 mm) [15], whereas other authors establish only two subgroups, pT3a and pT3b, based on a different cut‐off value ≥ 5 mm [16] or ≥ 3 mm [17], albeit the latter study analyzes together rectal and colon tumors.

The present study shows that the extent of extramural infiltration is an independent risk factor for LR (*p* = 0.04, HR 1.11 per mm), a higher risk of SR (*p* = 0.02, HR 1.08 per mm), worse DSF (*p* = 0.008, HR 1.08 per mm), and worse CSS (*p* = 0.009, HR 1.09 per mm). We decided to add this double cut‐off point at > 10 mm to explore the results of this group of patients with greater tumor infiltration. Although Merkel et al. [15] explored patients with > 15 mm in their study, we did not do so in our study due to the small number of patients with this depth. However, the sensitivity and specificity of this variable, when considered alone, are not high and should be evaluated alongside other significant factors when predicting the oncological outcomes in this group of patients.

Local recurrence in pT3 tumors is an important issue that has not been properly analyzed in any of the studies previously mentioned [15, 16, 17]. Importantly, the present study shows an evident prognostic heterogeneity effect of pT3 on local recurrence rates according to infiltration extent. In fact, the actuarial LR rate increases significantly from 4%, 12%, and 14% in relation to the depth of infiltration of ≤ 5, 5–10, or > 10 mm, respectively. In other words, the LR risk increases by 11% for each additional mm of depth infiltration.

Colon cancer surgery outcome depends not only on tumor stage but also on the quality of radical surgery [3, 30, 31]. Isolated LR rates can be considered a reflection of failure to perform adequate primary surgery, as it is in cases of rectal cancer, and thus are a measure of surgical expertise [28]. There is wide variation in rates of cumulative 5‐year local recurrence after curative colon cancer resection, ranging from 2.1% to 14.7% [2, 3, 26, 27, 28, 32, 33]. Thus, LR from colon cancer remains a significant clinical problem. In an institutional series of curative colon cancer surgery, the progressive application of the CME procedure resulted in a significant reduction of 5‐year LR rates from 6.5% in the period from 1978 to 1984 to 3.6% in 1995–2002, and an increasing cancer‐related 5‐year survival rate from 82.1% to 89.1%, respectively, in those periods [3]. Regarding the patients included in the present study (pT3M0), the actuarial LR rate is 6.3%, quite similar to the 5.2% described by Hohemberger et al. [3] However, other studies report higher pT3 LR rates, such as 11.4% [32] and 13.9% [2], showing the critical prognostic value of good quality surgery. There are several recognized independent risk factors for LR such as poor differentiation, higher T and N staging, vascular invasion, urgent surgery, and perforation [26, 28, 32], and for some authors, the different tumor colon locations [32, 33]. Nevertheless, it is remarkable, in the present analysis focused on pT3 tumors, that the extent of depth infiltration beyond the muscular layer is the only independent prognostic factor for local recurrence (*p* = 0.038).

The main limitation of the present analysis is that it is a retrospective study. Nonetheless, it includes a large institutional series of patients with a prolonged follow‐up in an MDT setting, audited by dedicated oncologists specialized in colorectal cancer. Second, since the long period of inclusion, an important percentage of patients had less than 12 lymph nodes examined. As previously described in a previous publication of our group [23], these were mainly patients at the beginning of the study period. Since the surgical technique was standardized among different colorectal surgeons, we would suggest that the improvement observed over time concerning the number of nodes retrieved in the specimen was likely to be because of a closer collaboration with the pathologists and their involvement in MDT, as well as because of a more specific training pathway in colon cancer pathologic examination and reporting setup during the last years for pathologists in our unit. Finally, as a limitation we should also mention that detailed data about adjuvant treatment such as complications and/or completeness were not included in the analysis.

In our analysis, the lack of effect of adjuvant chemotherapy may be due to a bias effect related to the selection by treating oncologists. Likewise, histological grades are not always statistically significant in other research papers [34]. They are not currently determinants in clinical practice to make therapeutic decisions, and other histological variants such as tumoral budding play a more important role. Histological grades usually show a lot of inter‐observer variability.

For pT3 rectal cancer, the last version of the TNM supplement proposed pT3a and pT3b subdivisions, based on not more than 5 mm or more than 5 mm beyond the outer border of the *muscularis propria* [19]. However, for pT3 colon cancer, the same TNM supplement proposed three different categories according to the extent of tumor invasion: pT3a (≤ 5 mm), pT3b (> 5–15 mm), and pT3c (> 15 mm). This proposal was based exclusively on differences in the 5‐year actuarial cancer‐related survival rates (89.8%, 85.8%, and 73.1%, respectively) found in the Erlangen Registry for Colorectal Cancer (1986–2003), without considering the findings from more recent studies on this subject [15, 16, 17].

The findings of this study are consistent with existing literature on rectal cancer, endorsing a 5 mm cut‐off as effective for stratifying the pT3 colonic cohort. However, the subdivision analysis based on infiltration depths (< 5, 5–10, and > 10 mm) proposed in this investigation represents a novel approach. This categorization holds promise for assessing prognostic variability within pT3 colonic adenocarcinomas, potentially enhancing predictive accuracy regarding oncological outcomes. Furthermore, it offers a rationale for tailored adjuvant therapy selection, particularly benefiting patients at heightened risk of adverse prognoses. Consequently, this study introduces a potentially significant tool for clinical decision‐making in the management of colorectal cancer.

In conclusion, the extramural spread in millimeters of pT3 colon cancer is a significant prognostic factor for worse oncological outcomes after curative colon cancer resection, this variable being more relevant in patients with positive lymph nodes. Our results are a clear appeal to the International Union Against Cancer/American Joint Committee on Cancer to include this updated new pT3 subdivision in the next TNM supplement or in the new edition of the TNM classification.

## Author Contributions

**Beatriz Arencibia‐Pérez:** conceptualization (equal), formal analysis (equal), investigation (equal), methodology (equal), supervision (equal), writing – original draft (equal), writing – review and editing (equal). **Francisco Giner:** conceptualization (equal), investigation (equal), methodology (equal), writing – original draft (equal), writing – review and editing (equal). **Eduardo García‐Granero:** conceptualization (equal), investigation (equal), methodology (equal), writing – original draft (equal), writing – review and editing (equal). **Susana Roselló‐Keränen:** conceptualization (equal), methodology (equal), writing – original draft (equal), writing – review and editing (equal). **Blas Flor‐Lorente:** conceptualization (equal), investigation (equal), writing – original draft (equal). **Andrés Cervantes:** conceptualization (equal), investigation (equal), writing – original draft (equal), writing – review and editing (equal). **Jorge Sancho‐Muriel:** conceptualization (equal), methodology (equal), writing – original draft (equal), writing – review and editing (equal). **Matteo Frasson:** conceptualization (equal), investigation (equal), writing – original draft (equal), writing – review and editing (equal).

## Ethics Statement

This study has been approved by the Ethical Committee of University Hospital La Fe of Valencia, and written informed consent was obtained from each patient.

## Conflicts of Interest

The authors declare no conflicts of interest.

## Data Availability

All data are available in tables and figures.
